# Examining the Contribution of Nurse Practitioners to Geriatric and Palliative Care in Israel

**DOI:** 10.3390/nursrep15030101

**Published:** 2025-03-15

**Authors:** Rachel Nissanholtz-Gannot, Keren Grinberg, Shoshy Goldberg, Hilla Fighel, Yael Sela, Yafit Cohen, Rivka Hazan Hazoref

**Affiliations:** 1 Department of Health Systems Management, School of Health Science, Ariel University, Ariel 4070000, Israel; rachelng@ariel.ac.il; 2 Smokler Center for Health Policy Research, Meyers JDC-Brookdale Institute, Jerusalem 9103702, Israel; yafitco@jdc.org; 3 Nursing Sciences Department, Faculty of Social and Community Sciences, Academic Center, Ruppin 4025000, Israel; kereng@ruppin.ac.il; 4 Nursing Division, Israeli Ministry of Health, Jerusalem 9101002, Israel; shoshy.goldberg@moh.gov.il (S.G.); hilla.fighel@moh.gov.il (H.F.); rivka.hazoref@moh.gov.il (R.H.H.)

**Keywords:** nurse practitioner, geriatric care, palliative care, health system policy, role perception

## Abstract

**Background/Objectives**: Nurse practitioners (NPs) play a pivotal role in delivering medical care, leveraging their specialized training and broader range of authorities than registered nurses, as approved by the Ministry of Health. Since 2009, Israel has expanded NP training to include diverse specialties such as palliative care, geriatrics, diabetes, surgery, and health policy. Introducing a new professional role into the health system is a complex, resource-intensive process that requires collaboration across stakeholders. Globally, NPs are recognized for preventing hospitalizations and achieving improved care outcomes, with high patient satisfaction. However, in Israel, NPs’ perceptions of their role and contributions remain underexplored. This study aims to assess NPs’ role perception, contributions to the health system, and attitudes toward their professional development in palliative and geriatric care. **Methods**: The mixed-methods study included 26 in-depth interviews with palliative and geriatric NPs and an online survey of 89 NPs (29 in geriatrics and 60 in palliative care). **Results**: Most NPs are women (84%), Israeli natives (69%), and Jewish (64%). More than half (53%) have 1–4 years of experience as practitioners, and 71% are employed full time. Regarding their work environment, the NPs feel that their supervisors and patients are appreciative of their work and that they are considered professional authorities. The qualitative findings also point to several challenges in their work: (1) The NPs’ status vis-à-vis medical staff and patients is insufficiently established; (2) the NPs lack practical and theoretical learning over time; (3) there is a shortage of positions; and (4) there is a sense of high work and emotional load and difficulty in implementing palliative care within the health system. With regard to their training, 58% of NPs were satisfied with the course, while 71% felt it lacked content. Looking forward, 76% of the NPs believed that over the next five years, the NP role would expand significantly, and 88% would recommend that other nurses become NPs. **Conclusions**: To maximize NPs’ potential, the study recommends clarifying their role, expanding authorities, aligning training with professional demands, and fostering trust between NPs and the medical establishment. Branding efforts and emotional support for NPs, especially in palliative care, are essential to enhance their integration and effectiveness in the health system.

## 1. Introduction

### Demographic Changes and Their Impact on the Israeli Health System

More than a million individuals aged 65 and older live in Israel today—12% of the population. This group is expected to increase to 14.7% by 2045 [1]. This change is accompanied by a trend of reducing the hospitalization of older adults in long-term care facilities (geriatric and nursing homes) and expanding community care services [2,3,4]. This trend is informed by the aging-in-place principle, which guides policies in many countries. In Israel, about 98% of older adults live in the community [1]; similarly, 87% of older adults in need of nursing care also live in the community [5]. According to the 1994 National Health Insurance Law, the health system is required to provide older and end-of-life patients appropriate health services based on the principles of justice, equality, and mutual support [6]. The provision of such services requires a trained and skillful dedicated care staff.

Despite the increase in life expectancy in Israel and the concomitant increase in the number of people with diseases that require monitoring by a doctor or a nurse practitioner (hereafter, NP) specializing in geriatric or palliative care, and despite the increase in personnel specializing in palliative care, studies show a persistent shortage in this area [7,8] and highlight that current training programs are inadequate for producing enough qualified professionals to meet the needs of the population requiring these services. A report from the Health Ministry on the National Program for End-of-Life Care [9] highlights several challenges in palliative care services, including slow service development, the absence of multidisciplinary consulting teams, uneven geographic distribution of nurse practitioners (NPs), and a disproportionate focus on providing palliative care services exclusively to cancer patients [10].

In the West, in the early 1960s, the University of Colorado opened the first academic NP program. The institutionalization of the profession began due to a shortage of primary care physicians. In the 1990s, the profession expanded to other countries, including Australia, the UK, and other European countries. Today, nurses with clinical expertise are considered an independent profession, with a recognized professional track clearly defining the training, certification, and employment conditions for NPs [11,12,13,14].

NPs have a broader range of authorities than that of registered nurses. These authorities are provided given the broader training NPs are required to complete. In the West, there is a trend toward broadening the scope of practitioner nursing by increasing both the number of NPs and the areas in which they can specialize (among other things, in the community). In 2018, 22 states in the US enabled NPs full practice authority, including diagnosis, treatment, and writing prescriptions, without medical involvement. Conversely, in the 28 remaining states, the NP’s authority is not enough, and medical involvement is required at a certain level [15]. States in the US also vary in terms of NPs’ work settings, role definitions, areas of practice, and authorities.

In Israel, the Ministry of Health began training NPs in 2009 following a special order that enabled the opening of a dedicated track for palliative care NPs. In 2013, with the promulgation of the Public Health Regulation, training in the geriatric area also became available, and specialty areas were added: diabetes, premature infants, surgery, community, health policy and administration, and pain. More recently, the areas of rehabilitation; injury, stoma, and incontinence; psychiatry; and pediatric emergency care were added.

The role of Israeli NPs is to provide medical care based on their training and the authorities bestowed upon them by the Director General of the Ministry of Health subject to their authority, outlined in Article 59 of the Physicians Order. This role has been modeled on the American role of the advanced nurse practitioner [16]. In addition to its contribution to the system, it is also seen as contributing to the nurse’s personal empowerment and professional development [17,18].

By the end of 2019, there were 275 NPs in Israel with clinical specialization certificates of various kinds; by the end of 2021, there were 453; by 2023, their number reached 730, and still, we anticipate a general shortage of nurses and positions in the future. This situation is referred to in the literature as a “care deficit”. This means that despite the rise in longevity and the percentage of older adults in Israel, the supply of formal personnel falls, and there is difficulty involved in recruiting new personnel for care professions [8].

NPs specializing in palliative care have been active in Israel since 2010. By 2024, they numbered 190 [19]. These NPs are employed in hospitals and in the community so that palliative care is provided both for hospitalized patients and those at home; it is provided not only to oncological patients but also to those with cardiac insufficiency and kidney failure, as well as to geriatric patients. The NP’s role in palliative care is highly diverse and includes examining the patients and assessing their condition; referrals to diagnostic and follow-up tests; formulating treatment plans and providing care instructions; symptom balancing, guidance and consulting for the patient and family; managing medicinal care, including onset, balancing, and termination; providing unique treatments recognized as belonging within the NPs’ authority; involving relevant care providers in community care; and direct end-of-life care. In fact, the palliative care NP is the patient and family’s backbone, responsible for coordinating all care providers and maintaining an ongoing dialogue between all members of the care staff [20].

NPs specializing in geriatric care have been active in Israel since 2013, subject to the Public Health Regulations. They began working in geriatric medical centers [21]. By 2023, their number reached 144 [22,23]. A Ministry of Health circular updated their work procedures, and it was decided to expand their employment to general hospitals and geriatric consulting. The job of a geriatric NP therefore includes two areas: providing direct care to older adults in hospitals and providing geriatric consulting in the community [21].

Internationally, the NP role has evolved differentially. In the United States, for instance, 22 states permit full autonomous practice for NPs, including prescription authority without physician oversight [22]. In the Netherlands, a recent study by [23] demonstrated that NPs in outpatient care significantly improved access to care and patient satisfaction. These international variations provide crucial context for understanding the development of the NP role in Israel.

As in many other countries, the increasing longevity and the rise in complex chronic morbidity pose real challenges to the Israeli system that threaten its ability to function effectively. It is therefore highly appropriate to examine the perceptions of NPs specializing in palliative and geriatric care regarding their unique contribution to the health system and their community (or where such a contribution could be made) and examine how they believe the NP’s role should be developed in these areas, based on their current contribution and future courses of development.

The overarching objective of this study was to examine the way NPs and policymakers in the Israeli health system perceive their contribution to the system and the potential development of their roles in the palliative and geriatric care areas. The study’s secondary objectives were as follows:

1. To examine the perceptions of palliative and geriatric care NPs regarding their unique contribution to the health system and where this contribution can be made, as well as the differences between the groups.
2. To examine how the nurses believe their role in these areas can be developed.

## 2. Materials and Methods

The study was approved by the Myers-JDC-Brookdale Ethics Committee, under approval number IRB-BH041. All participants provided informed consent prior to their involvement in the study. The consent process included information about the study’s purpose, the voluntary nature of participation, the right to withdraw at any time without consequences, and the confidentiality measures used to protect the participants’ privacy.

This mixed-methods study combined quantitative and qualitative methods. In the first stage, eleven semi-structured interviews were conducted with six geriatric and five palliative care NPs. Participants were required to be licensed NPs actively working in geriatric or palliative care. Retired NPs and those not involved in direct patient care were excluded. The snowball sampling method was initiated by contacting senior NPs, and efforts were made to include professionals from diverse healthcare settings, such as hospitals, community clinics, and nursing homes, to ensure a broad range of perspectives.

An attempt was made to select respondents whose experience and work environments differ to maximize variance and allow for a variety of perspectives and lived experiences. The interviews were conducted using a semi-structured interview guide, which included questions on the contributions of geriatric and palliative care NPs, on ways to develop the NP role, on the professional training provided to NPs, and on their authorities and role challenges. Peer debriefing was conducted as part of the validation process. Discussions with colleagues helped critically assess and refine the interpretations, ensuring credibility and reducing potential biases. The interviews (approx. 90 min) were conducted from January 2019 to July 2021, face to face or over the phone, according to the interviewee’s preference. The first three interviews were conducted as a “pilot”, helping to direct and focus the questions planned for the remaining interviews on the most important and relevant issues. Next, additional interviews were conducted based on the insights gained from the pilot interviews.

In the second stage, an online self-report survey was conducted among all geriatric and palliative care NPs in Israel (*N* = 119), based on a list provided by the Ministry of Health. The survey form was emailed to 101 participants, after 8 were removed from the list, mostly because they had already retired. Of these, 10% were excluded after refusing to participate or failing to respond. Another 2% were removed because they did not complete the entire survey. All participants completed the survey themselves. The final sample of 89 participants represented NPs of both specialties—geriatric and palliative care—with diverse levels of experience and backgrounds. The quantitative research instrument—the online survey—included 90 questions, mostly closed-ended, in six areas: demographic and occupational characteristics; the content of their job and their functioning on the job; authorities and autonomy; professional training; the status of the NP; and views regarding the future of the profession. The survey was constructed based on a review of the literature and on the findings of the in-depth interviews conducted in the first stage.

### Data Analysis

The interviews were analyzed thematically in several stages [24]. First, they were transcribed and reread repeatedly to identify emerging issues. In the second stage, the text was broken down into units of meaning serving as basic analytical components. Open coding was conducted to identify key concepts, which were then grouped into preliminary categories. Through an iterative process, these categories were refined and developed into overarching themes. Thematic analysis involved constant comparison between transcripts to ensure consistency and depth. Adjustments were made throughout the process to refine themes, ensuring they accurately represented the data. The final themes were determined based on their relevance to the research question and supported by representative quotes.

In the following stage, the main categories were extracted and formulated into themes. The last stage involved conceptualizing the links and relations between the themes. The main themes are presented in the Results section. For quantitative analysis, we employed SPSS version 26 software. Descriptive statistics were utilized to characterize the sample and response distributions. Independent *t*-tests were conducted to compare means between the geriatric and palliative NP groups for the continuous variables, while chi-square tests were used for categorical variables. Statistical significance was set at *p* < 0.05. Additionally, effect sizes (Cohen’s *d*) were calculated for the significant differences observed.

## 3. Results

### 3.1. Demographic and Occupational Characteristics

Twenty-nine of the NPs who participated in the survey specialized in geriatrics, and sixty specialized in palliative care (see Table 1). Most (84%) of the participants were women; in geriatric care, the rate of male nurses was higher than in palliative care (31% vs. 8%). The mean age of all NPs was 53. Two thirds (69%) were Israeli natives: there were more natives among the palliative care NPS than among the geriatric care NPs (75% vs. 59%). Most (88%) of the participants were Jewish, and 64% from the total sample were secular. In terms of professional experience, 80% of the NPs had worked as nurses for more than 20 years; as NPs, 53% had 1–4 years of experience, 37% had 5–10 years of experience, and 11% had more than 10 years of experience. Half (48%) of the geriatric specialists were actually employed as NPs, as opposed to 73% of the palliative care specialists. Out of all the NPs, 71% were employed full time; 93% of the geriatric specialists were employed full time, as opposed to 64% of the palliative care specialists.

The participants’ occupational characteristics are presented in Table 2. Clearly, NPs are experienced nurses, and most are employed full time and in a variety of settings.

### 3.2. The Nurse Practitioners’ Reported Activities

**Figure 1:**
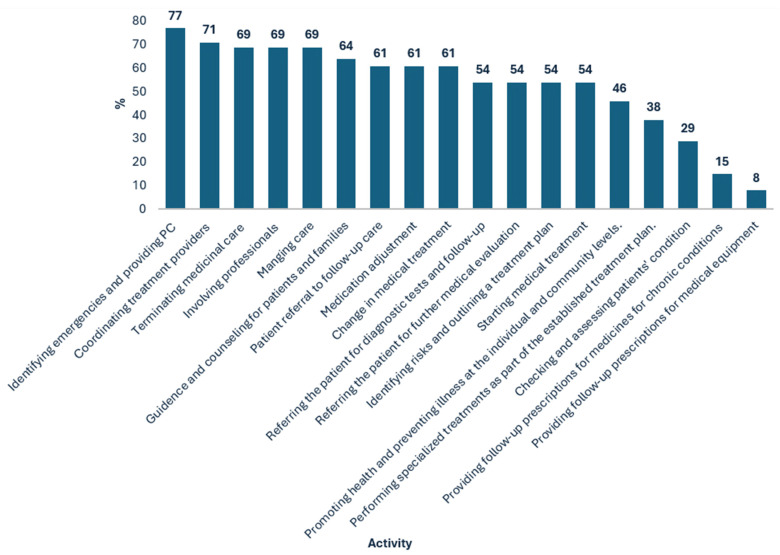
Reported Geriatric Nurse Practitioner Activities (in %).

**Figure 2:**
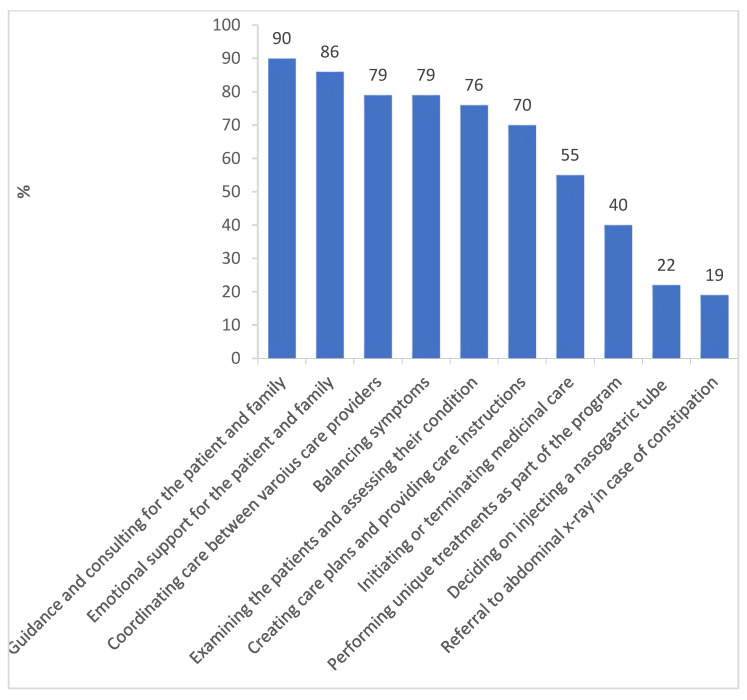
Reported Palliative Care Nurse Practitioner Activities (in %).

The Ministry of Health [22] published a list of activities included within the authority of NPs, as shown in Figure 1 and Figure 2 for geriatric and palliative care, respectively. The percentages in those figures indicate the degree to which each activity is included in the NPs’ scope of responsibility. It appears that in the geriatric area, the activities that take up a larger part of the NPs’ work include identifying emergencies and providing primary care (77%), coordinating treatment providers (71%), involving professionals (69%), and terminating medicinal care (69%). An activity that the nurses reported hardly doing was providing follow-up prescriptions for medicines for chronic conditions (85%).

In the palliative care area, the activities that take up a larger part of the NPs’ work were reportedly providing guidance and counselling to the patients and their family (90%), providing emotional support to the patients and their family (96%), treatment coordination (79%), symptom balancing (79%), examining the patients and assessing their condition (76%), and setting up treatment plans and providing treatment instructions (70%). Two activities that the nurses reported hardly doing at all were referral to abdominal X-rays in cases of constipation (19%) and deciding on injecting nasogastric tubes (22%).

### 3.3. The Nurse Practitioners’ Fourfold Impact

The added value of the NPs’ role is expressed in six main areas: symptom balancing (88%); contribution to training and staff development (81%); ability to provide supervision (77%); greater authorities compared to registered nurses (77%); maintaining and promoting treatment continuity (72%); and providing holistic treatment (68%). Conversely, only 19% of the NPs saw added value in the time they spent on preventing morbidity. Overall, the NPs believed their role to be highly necessary: 98% replied that the NP role was highly necessary; 87% felt that they performed their job effectively, to a considerable degree.

The in-depth interviews suggested that the NPs’ contribution impacted four circles: the patient, the family members treating the patient, the professionals, and the health system. It was also found that specialization benefited the nurses themselves by providing a career horizon, and that it may prevent attrition by enabling nurses to treat the specific populations they are interested in treating (Table 3).

#### 3.3.1. Circle of Influence 1: The Patient

1. Preventive medicine is considered essential as it can protect patients against acute conditions, hospitalization, and the deterioration of their condition and improve their quality of life. The NPs’ skills enable them to assess patients’ situations before they deteriorate and thereby improve their health and prevent morbidity. Some of the nurses even consider this a central part of their work, although they lack the time to devote themselves to it.

*I can also evaluate the patient on my own […]. In fact, I’m working on it write now, patients aged 65–75 who are still healthy without repeated hospitalizations […] still high functioning.*

*(Interviewee #10, geriatric NP)*

2. Holistic view of the patient. The NP is capable of viewing the patient holistically, considering not only the strictly medical aspect, but also others, such as the family system and socioeconomic situation. This holistic view contributes to the patient’s quality of life and to the quality of the medical care, including prevention, institutionalization, and hospitalization, since it contributes to formulating a customized and optimal treatment program.

*The mandate of an NP is truly comprehensive, including the physical, familia, and social aspects, and the broader characteristics… You look at the next step, where he goes from here, whether it’s to the community or to hospitalization. I am constantly preoccupied with predicting the next challenges that would have to be dealt with and with trying to provide a far more comprehensive response, which is medical, and also social, and also emotional. And that is something that an ordinary nurse does not have the time and usually not the ability [to do].*

*(#9, palliative NP)*

3. Ability to treat complex and extreme cases. The interviewees suggested that NPs can devote time to treating complex and extreme cases that ordinary nurses find difficult to cope with. According to one of the interviewees, the NP is supposed to treat only the complex cases—about 5% of all patients, and in fact, they become the patients’ treatment managers, covering all required aspects.

#### 3.3.2. Circle of Influence 2: The Family

The NPs’ position enables them to maintain close and ongoing contact with the family members treating the patient, with higher availability than that of the responsible doctor, thus improving the quality of care:

*Today, in order to talk to a doctor, the family members have to wait for a long time… An NP can contact the family to provide them with answers, because she’s in a fulltime position, and that’s very important. The family needs to know that there’s a professional, with superb professional level, not just good, who can provide clear and serious answers. That will reduce much of the families’ disquiet.*

*(#19, nursing home manager)*

#### 3.3.3. Circle of Influence 3: Professionals

Medical authority and professional knowledge. NPs have the requisite training, experience, and knowledge, which they continue to accumulate on an ongoing basis. Therefore, they provide doctors and colleagues with another professional authority for consultation.

Reducing the doctors’ workload is another important contribution of the NPs, seeing that they are able to make decisions ordinary nurses cannot:

*The NP is actually the doctor’s substitute in daily life… She has good diagnostic skills and her shoulders are wide enough… She is the one who can help in the nursing area, and also make the right semi-medical decisions.*

*(#19, nursing home manager)*

#### 3.3.4. Circle of Influence 4: The Health System

The NPs’ contribution to the health system is derived from their contribution to the patients and their families, as well as their contribution to the professionals, enabling them to enhance the performance of the entire system. This contribution is expressed in two areas, as follows.

Treatment coordination. The NPs specialize in geriatrics and palliative care, enabling them not only to contribute individually to patients and other professionals, but also to coordinate the entire treatment plan.

Reducing hospitalization and institutionalization rates. The NPs’ work (especially in the community) can help prevent hospitalization and institutionalization, since NPs can monitor at-risk populations and initiate preventive actions:

*The NP will be able to follow up on them… In every clinic you have elderly patients with chronic illnesses, and if I want to take all these groups and monitor their illnesses and reach out to them and maintain their quality of life, I can save lots of hospital days.*

*(#20, nursing school lecturer)*

### 3.4. The Nurse Practitioners’ Status

The NPs’ ability to contribute and make the most of their abilities is also influenced by the profession’s status in the health system. The survey and interviews indicate that this status is influenced by three main factors: (1) Identity—professional categorization and the construction of a professional identity between nursing and medicine. (2) Professional performance, including (a) autonomy, authorities, and responsibilities as opposed to professional subordination; (b) national formalization as opposed to local initiatives. (3) Environment—the NPs’ work environment and the interface between their roles and those of other professionals.

#### 3.4.1. Identity

A key aspect in the NPs’ role that affects their status is their perception regarding their professional categorization. The respondents were asked whether they felt they belonged in the medical or nursing sector. The geriatric NPs felt that they belonged in the medical sector more than in the nursing sector (71% vs. 50%; Cohen’s *d* = 3.4, *p* < 0.001). The palliative care NPs, on the other hand, felt more part of the nursing staff than the medical staff (90% vs. 50%; Cohen’s *d* = 3.8; *p* < 0.001).

The interviews also pointed to ambivalence regarding the NPs’ professional categorization. They also indicated that this ambivalence, and the fact that each sector tried to pull the NPs to its side, could lead to a confused professional identity and role definition:

*[I’m] on the fence—one foot here, one foot there. I don’t see myself as belonging to either sector, “purely”. I cannot not be a nurse and see the nursing aspect, but I no longer view it strictly as a nurse, I only add the extra aspect… On the whole, I consider myself part of the medicine thing, because that’s what’s expected of me in the role definition… I sit in the doctors’ lounge, I don’t sit in the nursing”.*

*(#4, geriatric NP and senior manager)*

Another aspect of belonging to a certain sector is professional subordination. On the one hand, the NPs are subordinate to the doctors with whom they work; on the other hand, administratively, they belong in the nursing sector. This creates additional confusion:

*Our system is very hierarchic […]. They’re creating a hybrid creature here—the nurse is subordinate to the doctor under whom she works, but the administrative subordination is to nursing, and this creates difficulties.*

*(#24, doctor in the community)*

#### 3.4.2. Professionalism

One of the aspects most relevant to the NPs’ status is their scope of authorities, autonomy, and responsibilities. A recent Director General circular granted geriatric NPs medical authorities previously denied to NPs in Israel [23]. The authorities added to their role included independent diagnosis of multiple medical conditions; the authority to refer to the entire medical examination and consultation system, to health professions, and to auxiliary examinations, without need for examination or approval by the family doctor; and partial authorities for treatment and prescription. The nurses were asked whom they reported to, who determines their schedule, who determines the nature of their patients, who is professionally responsible for them, how autonomous they feel, and to what extent their status as a professional authority has changed since becoming an NP. Their responses (from both geriatric and palliative care NPs) were as follows:

- Among the NPs, 70% reported their activity to the unit’s medical manager, and 53% to the nursing manager of their hospital or organization; in other words, there were quite a few instances of “multiple reporting”.
- Meanwhile, 24% of the NPs reported being subordinated to a single professional individual/entity in the system, 28% reported being subordinate to two, and 9% to three, while 1% even reported being subordinate to four.
- Among the nurses, 37% reported that one professional individual/entity decided on the identity of their patients, 18% reported two, 3% reported three, and 2% reported four.

### 3.5. Challenges in the Nurse Practitioner’s Role

#### 3.5.1. Establishing the Nurse Practitioner’s Status

This section outlines the challenges raised by the participants with regard to coping with resistance of the medical establishment, the NPs’ role definition and expanded authorities, coping with labor relation management, working under the supervision of department directors, the need to prove professionalism, the need to have the profession recognized by hospitals, and dealing with prejudice towards their role. Establishing the NPs’ status also includes the need to raise patients’ and the general public’s awareness of their profession, to prevent a situation where patients and their families prefer not to receive medical treatment from nurses but only from doctors:

*Another challenge is the public. They want doctors. They come for the department director, and that’s a difficult challenge.*

*(#20, nursing school lecturer)*

The feeling that the medical establishment is opposed to the NPs’ role and expanded authorities was mentioned by nearly all interviewees as an essential and critical issue. The resistance seems to be led by the Israel Medicine Association and involves both doctors and pharmacists. The NPs’ experience its manifestations in various ways and explained it mainly in terms of the pharmacists’ and doctors’ fear of losing status and authority. From their perspective, the very idea of an NP profession is objectionable, since they fear the quality of care will be affected by the lack of sufficient skills and the need to save on doctors’ positions:

*I’m against the idea of practitioner nurses, because here in Israel the objective is not to make life easier for the doctors, but to avoid adding doctors’ positions and letting nurses do the doctors’ work… How? At the expense of the patients. Our patients are old, they cannot shout that they want quality treatment, so it’s easy to provide them treatment with less-skilled staff.*

*(#23, doctor and geriatric department director)*

#### 3.5.2. Training

The nurses divided the training issue into two issues: (1) The need to expand theoretical knowledge, including reviews of recent professional literature, setting quality standards for the NPs’ work, and integrating academic knowledge into daily practice; and (2) the need to expand their practical knowledge, including learning methods such as holistic care, and promoting the palliative care approach.

#### 3.5.3. Positions

One of the challenges of the health system is the shortage of NP positions. Both the NPs and other professionals reported this to be one of the profession’s main challenges, a barrier preventing it from developing and expanding. Although it is agreed that adding NP positions would make up more time for doctors by reducing their workload and improve the availability of services in the system, the NP positions available are still insufficient.

#### 3.5.4. Workload

The nurses described two related issues: (1) Time management and resource allocation between the patients—the need to prioritize treatments; and (2) emotional burden and a sense of burnout due to the nature of their role: supporting patients and families in end-of-life situations and coping with the loss of patients. This was also found in the survey: the NPs in palliative care experienced significantly more emotional difficulties than their geriatric counterparts (40% vs. 14%) and were also significantly more interested in emotional support (51% vs. 14%; *p.* < 0.05).

#### 3.5.5. Integrating Palliative Care into the Health System

This challenge included (1) the integration of the role; and (2) the integration of both the approach and the care itself, since this is not yet available in all hospitals and community health services. The palliative care NPs suggested that awareness of this area had to be raised among doctors in hospital departments; that the palliative care approach had to be integrated even in non-oncological departments; that resources had to be provided for the establishment of a comprehensive palliative care system; that multidisciplinary staff needed to be integrated; and that the treatment provided in home hospices needed to be improved.

It seems that most of the challenges raised by the nurses need to be addressed at the systemic level. Some challenges are interrelated, such as the high workload and need for additional positions. The challenges of establishing the NPs’ status and expanding their clinical knowledge should be addressed by the Nursing Administration and serve as a basis for future action.

### 3.6. The NPs’ View of the Future of Their Profession

The participants were asked about their views regarding the future of the NP profession, from a variety of aspects. They were highly pleased with their present role (96%). Among the NPs, 23% expressed a desire to change role within the health system, and only 8% expressed a desire to leave the health system. Nevertheless, 23% expressed a desire for professional change within the system.

Three quarters (76%) of all NPs believed that within five years, their role would expand. Many more of the geriatric NPs thought this than palliative care NPs (93% vs. 71%). Most (88%) of the respondents would recommend that other nurses join the profession, for a variety of reasons: there is need for more NPs; it is a challenging, satisfying, and meaningful profession; the profession contributes to the patient; the profession contributes to the nurse’s professional development and empowers her to realize her potential as a professional; finally, it is important for every hospital ward to have such a nurse given their unique contribution. The differences between the two specialty areas were nonsignificant in these regards (*p* < 0.05).

### 3.7. Crosscutting Theme: Role Ambiguity

The interviews indicate that all the themes that arose in the findings are foregrounded by a shared meta-theme: role ambiguity. This ambiguity is characteristic of all aspects of the NP profession: role definition, status, authorities, responsibilities, autonomy, subordination, and professional categorization.

## 4. Discussion

This study examined the contributions of nurse practitioners (NPs) in order to suggest potential paths for their future development as a profession, with an emphasis on NPs specializing in geriatric and palliative care. The findings indicate that the NPs make important contributions in four circles of influence: the patient, family caregivers, professionals, and the health system. An additional benefit of their specialization is for themselves, as it can prevent burnout and attrition among nurses and opens up possibilities for promotion and diversification in the activity area, for a changed work environment, and for a focal influence on the population the nurse is interested in caring for.

The contributions of the NP have already been examined in the literature, with studies showing that NPs improve the care of marginalized populations and prevent hospitalizations [25,26]. They also contribute to monitoring chronic patients, such as diabetics, at a level and with clinical results identical to those of monitoring by physicians [15,26]. Finally, a comprehensive review of the literature by Lovnik et al. (2017) found that in terms of treatment process and outcomes, NPs achieve results that are at least as good as those achieved by doctors [27].

Nevertheless, the findings point to ambiguity in the NPs’ role definition, its boundaries, and the authorities it includes. This ambiguity is all the greater since the nurses’ sectoral categorization and professional subordination are split between the medical and nursing worlds. At the same time, note that the present study indicates that this ambiguity enables the NP to engage in a wide range of activities and adjust them to the patient’s needs following the principle of “patient at the center”. The wide range and maneuverability enabled by the role provide the NPs with significant advantages in providing care and in conducting themselves in the professional arena.

The findings also point to a gap between the NPs’ training and their sphere of action. It appears that not relying on the knowledge provided by the NP training can affect all her circles of influence, as well as her job satisfaction. A study on palliative care NPs in Israel found that nurses’ satisfaction is related to four factors: (1) their relationship with other staff members; (2) doctors’ professional evaluation; (3) the ability to minimize medical complications; and (4) the degree of role autonomy [28]. These findings also highlight the importance of relationships with doctors and colleagues.

The issue of the NP’s role definition, autonomy, preparatory training, and boundaries has also been discussed in other countries [29]. The ambiguity of the NP’s role was studied in the Netherlands [30]. In the US, it was argued that administrative regulations restricted the NP’s independence [22,31]. A systematic review of studies on NPs recommended abandoning regulations and policies preventing the NPs from providing the complete range of medical service for which they have been trained, since their activity has a positive effect on treatment results and even reduces its costs [22,30]. Finally, it was found that increasing the NP’s autonomy could contribute to their retention [31,32,33].

The nurses feel a high degree of autonomy in their role, but only some of them feel that their status as professional authorities has changed with the transition to the NP role. NPs in the community feel less autonomous than nurses in institutions, and a greater number are considering leaving the profession. International studies suggest that NPs, particularly in geriatric and palliative care, contribute to reducing hospital admissions, enhancing care continuity, and providing more personalized attention to patients. However, the ambiguity of the NP role in Israel, regarding both authority and training, mirrors challenges seen in other countries, such as the United States and the Netherlands, where NPs often struggle with role clarity and professional recognition. The study emphasizes the importance of increasing NP autonomy, a recommendation shared across multiple healthcare systems worldwide.

### 4.1. Conclusions and Recommendations

The NP role is a profession in the making, with multiple significant contributions. Regulations should focus on promoting collaboration between NPs and physicians, strengthening inter-professional relationships to enhance patient outcomes. These policy changes could contribute to increasing the number of NPs in Israel, meeting the growing demands of an aging population, and improving the quality of geriatric and palliative care. We therefore recommend continuing to follow its development and assimilation and also examining the potential integration of NPs in additional areas, with emphasis on community work and on being an essential part of the health system.

In order to continue developing the NPs’ role and confront the challenges mentioned above, we recommend the following courses of action:

1. Providing a more precise definition of the NPs’ role and regulating their authorities.
2. Aligning the NPs’ professional training with job requirements according to role factors, such as:
  - Content expertise—knowledge on the content and subject of the role;
  - Skill—mastery of skills required to perform the role and acquirement of the relevant toolset;
  - Role identity—with emphasis on the NPs’ work in the community and developing abilities for ongoing knowledge refreshment.
3. Strengthening the relationships and trust between the medical establishment and the NPs through dialogue and opportunities for professional encounters, such as conferences and peer forums for consultation.
4. Emphasizing emotional support for palliative care NPs.
5. Branding the NPs’ role using special badges and logos to highlight their uniqueness in the medical system.

#### Implications for Health Policy

Our findings have significant implications for health policy in Israel. Primarily, the expansion of the NP role could alleviate physician shortages in geriatric and palliative care areas. We recommend that policymakers consider the following:

1. Increasing the number of training positions for NPs in geriatrics and palliative care.
2. Developing clear guidelines for autonomous NP practice, especially in community settings.
3. Implementing continuing education programs to maintain NPs’ skill currency.
4. Creating financial incentives to attract more nurses to NP specializations.

These steps will contribute to attracting more nurses to the NP role and at the same time to reducing the resistance of the medical establishment to expanding their authorities. These two results are essential given the aging of the population, the growing importance attributed to the principle of “aging in place”, and the increased demand for both geriatric and palliative care. Knowledgeable and experienced NPs can increase the quality and availability of the services provided in clinics and hospitals, contributing to both patients and their families and to the entire medical staff and improving the ability of the entire healthcare system to meet its growing challenges.

This study has several limitations. First, the sample size for the qualitative phase was relatively small, and participants were selected using a snowball technique, which may have introduced selection bias. Second, the study focused solely on geriatric and palliative care NPs in Israel, limiting the generalizability of the findings to other specialties and healthcare systems. Third, while efforts were made to ensure a diverse sample, the study may not fully capture the perspectives of all NPs, particularly those working in different institutional settings or with varying levels of experience. Fourth, the survey relied on self-reported data, which could be subject to response bias. Finally, the study did not include perspectives from other healthcare professionals or patients, which could provide a more comprehensive understanding of the NP role and its impact.

## Figures and Tables

**Table 1:** Background Characteristics of Nurse Practitioners (in %).

	Total	Geriatrics	Palliative
Respondents			
*n*	89	29	60
%	100	33	67
Gender			
M	16	31	8
F	84	69	92
M_age_	53	52	55
Birthplace			
Israel	69	59	75
Other	31	41	25
Nationality/Religion			
Jewish	88	86	88
Muslim Arab	8	10	7
Christian Arab	3	3	3
Other	1	0	2

**Table 2:** Occupational Characteristics of Nurse Practitioners (in %).

	Total (*N* = 89)	Geriatrics (*n* = 29)	Palliative (*n* = 60)
Experience as nurse (in years)			
10–20	20	28	17
21–30	38	37	38
31–50	42	35	45
Currently employed as NP	65	48	73
Experience as NP (in years)			
1–4	53	43	56
5–10	37	57	30
10+	10	-	14
Employment scope			
Part time	29	7	36
Full time	72	93	64
Managerial role	53	7	67
Workplace			
Dept. in general hospital	9	5	7
Consulting service in general hospital	26	-	35
Dept. in geriatric hospital	22	64	-
Consulting service in geriatric hospital	2	-	4
Hospital ambulatory clinic	2	7	-
Primary clinic in the community	4	-	5
Specialist clinic in the community	4	7	2
Consultation clinic in the community	2	-	7
Home visits in the community unit	2	-	7
Home hospitalization in the community	5	-	5
Hospice in hospital	4	-	5
Home hospice	-	-	-
Managerial staff unit/administration	18	17	23
No. of patients per week (full time)			
5–10	20	-	30
10–30	38	62	26
+30	33	39	30
Not providing direct care	10	-	15

**Table 3:** Summary of the Nurse Practitioners’ Unique Contributions.

Stakeholder	Contribution
Patient	Preventive medicine Holistic view of the patient Ability to treat complex and extreme cases
Family members	High availability
Professionals	Authority on medical consulting Reducing the physicians’ workload
Health system	Coordinating care Reducing the rates of hospitalization/institutionalization

## Data Availability

Data supporting reported results are available from the corresponding author upon request.

## Public Involvement Statement

There was no public involvement in any aspect of this research.

## Guidelines and Standards Statement

This manuscript was drafted against the Consolidated criteria for reporting qualitative research (COREQ) for the qualitative data, and for the quantitative data, the study used the Strengthening the Reporting of Observational Studies in Epidemiology (STROBE).

## Abbreviations

The following abbreviations are used in this manuscript:

NP	Nurse practitioner
